# Blended learning approach improves teaching in a problem-based learning environment in orthopedics - a pilot study

**DOI:** 10.1186/1472-6920-14-17

**Published:** 2014-01-27

**Authors:** David A Back, Nicole Haberstroh, Andrea Antolic, Kai Sostmann, Gerhard Schmidmaier, Eike Hoff

**Affiliations:** 1 Department of Traumatology and Orthopedics, German Armed Forces Hospital Berlin, Scharnhorststrasse 13, 10115 Berlin, Germany; 2 Dieter Scheffner Center for Medical Teaching and Educational Research, Campus Virchow Klinikum, Charité – Universitätsmedizin Berlin, Augustenburger Platz 1, 13353 Berlin, Germany; 3 Center for Musculoskeletal Surgery, Department of Orthopedics, Charité – Universitätsmedizin Berlin, Berlin, Germany; 4 Reformed Medical Track Program, Charité – Universitätsmedizin Berlin, Berlin, Germany; 5 Dieter Scheffner Center for Medical Teaching and Educational Research, Charité – Universitätsmedizin Berlin, Berlin, Germany; 6 Department for Orthopedics, Traumatology and Paraplegiology, University of Heidelberg, Heidelberg, Germany; 7 Julius Wolff Institute and Berlin-Brandenburg Center for Regenerative Therapies, Charité – Universitätsmedizin Berlin, Berlin, Germany

**Keywords:** E-learning, Orthopedics, Traumatology, Problem-based learning, Blended learning

## Abstract

**Background:**

While e-learning is enjoying increasing popularity as adjunct in modern teaching, studies on this topic should shift from mere evaluation of students’ satisfaction towards assessing its benefits on enhancement of knowledge and skills. This pilot study aimed to detect the teaching effects of a blended learning program on students of orthopedics and traumatology in the context of a problem-based learning environment.

**Methods:**

The project NESTOR (network for students in traumatology and orthopedics) was offered to students in a problem-based learning course. Participants completed written tests before and directly after the course, followed by a final written test and an objective structured clinical examination (OSCE) as well as an evaluation questionnaire at the end of the semester. Results were compared within the group of NESTOR users and non-users and between these two groups.

**Results:**

Participants (n = 53) rated their experiences very positively. An enhancement in knowledge was found directly after the course and at the final written test for both groups (p < 0.001). NESTOR users scored higher than non-users in the post-tests, while the OSCE revealed no differences between the groups.

**Conclusions:**

This pilot study showed a positive effect of the blended learning approach on knowledge enhancement and satisfaction of participating students. However, it will be an aim for the future to further explore the chances of this approach and internet-based technologies for possibilities to improve also practical examination skills.

## Background

In recent years, there has been a growing interest in research on the education and learning progress of students in medicine. Great effort has been put into knowledge transfer via internet-based electronic learning (*e-learning*) [1].

E-learning has also become an integral part of medical education [2-10]. Various authors have shown that greatest benefit and student satisfaction is achieved when combining e-learning with face-to-face courses, as *blended learning*[2-4]. Blended learning comprises the systematic integration of online and face-to-face engagement to support and enhance a meaningful interaction between students, teachers and resources [11,12]. When attempting to successfully facilitate the transfer of knowledge, it is essential that teaching be competent, appealing, and recipient oriented [5]. In this context, e-learning can be achieved e.g. by providing videos [6], podcasts [7] or interactive diagnostic tools [8], leading to a considerable improvement of knowledge transfer capabilities in a mix with face-to-face lessons [9,10]. However, more studies are still needed to proof the impact of e-learning and blended learning on the enhancement of students’ knowledge and clinical skills in general and in the field of orthopedics and traumatology particularly.

After the launch of e-learning courses in recent years, many studies have focused primarily on evaluating students’ satisfaction – an important factor in acceptance and use of e-learning [13-15]. Others have additionally analyzed e-learning’s influence on the acquisition of knowledge or skills [3,6,8,9,12,16].

In traumatology and orthopedics education such studies are comparatively still rare [13,17,18], despite the fact that e-learning might substantially improve quality and success of teaching in these highly clinically and practically oriented disciplines. While the number of musculoskeletal diseases and injuries has been steadily increasing over the last years [19], data indicate that medical student education concerning the diagnosis and treatment of musculoskeletal diseases should be enhanced [20]. Here, e-learning could add appeal [21] and promote better knowledge and clinical skills [18] by providing useful multimedia adjuncts (e.g. videos, podcasts, or radiological cases). However, more data is still needed to guide the design of blended learning curricula in these subjects, questioning especially to what extend the use of e-learning might be beneficial and to investigate the effect of different approaches or configurations of e-learning.

To further address these issues, we performed a pilot study in the teaching of students in orthopedics and traumatology. The chosen curriculum focused on strengthening interdisciplinary knowledge and heavily utilized problem based learning (PBL) with a student-centered teaching approach, encouraging problem-oriented, self-directed and self-organized learning. To evaluate different aspects of incorporating a supplementary e-learning component, following questions were asked in this study:

1. Will students appreciate an additional e-learning offer in a blended learning context?

2. Will user of the e-learning offer show a superior improvement in theoretical knowledge compared to non-users?

3. Will users perform better in clinical examination skills compared to non-users?

The findings should allow a more informed discussion about the aspects that may have to be considered when integrating blended learning approaches into a PBL curriculum of orthopedics and traumatology.

## Methods

### Setting

**Figure 1:**
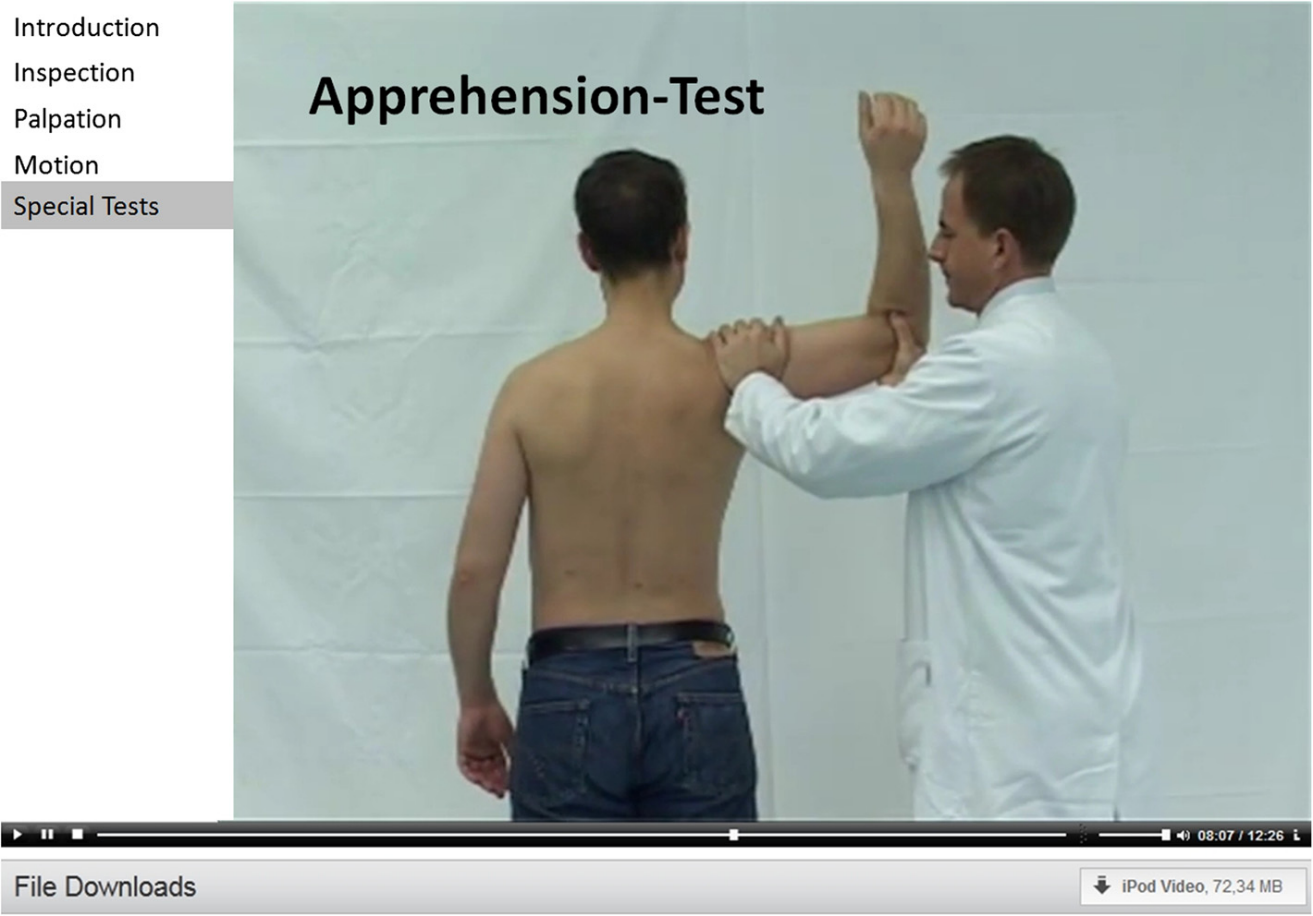
**Examination video.** Legend: Example of an examination situation of the shoulder (here: Special tests – Apprehension-Test) as shown in the videos on NESTOR.

All students taking part in the pilot study were in their fifth year of medical studies in winter semester 2009/ 2010. In their curriculum, for the first five of six years (two semesters per year) teaching was organized into block courses, covering different topics along a longitudinal learning helix. Following basic orthopedic concepts in the first semester, a two week block course, “Upper Extremities and Spine”, was specifically dedicated to increase knowledge of traumatologic and orthopedic diseases and furthering clinical skills in the ninth semester. Participation in the latter course was mandatory for all students and this course was chosen to incorporate a new e-learning module called NESTOR (network for students in traumatology and orthopedics), provided through the learning management system (LMS) *Blackboard* (Blackboard Inc., Washington D.C., USA) with multiple features:

–Orthopedic examination videos (covering inspection, palpation, motion and special tests) (Figure 1).

–Interactive radiology cases with X-rays, MRI- or CT-images and a patient history. After being asked to generate and enter a hypothesis for the diagnosis, the correct answer was given along with explanations for the ensuing treatment.

–Audiovisual podcasts for common traumatologic or orthopedic diseases (with medical history, diagnostics, therapy, and prognosis).

–Multiple-choice questions were available all the time to enable the students to self-test their gain in knowledge.

### Design of the study

Prior to the beginning of the course, students were informed where to find and how to access NESTOR on the LMS Blackboard and about its contents. Clinical tutors were provided with similar information. All students of the semester who were as well participants in the course were asked to take part in the study. The option of enrolling in NESTOR was voluntary. All participants had continuous access to NESTOR during the whole semester. The e-learning module contained no information not otherwise taught (e.g. in classes or regular study books). A tracking function to detect the individual accessed e-learning tools or the time students spend with them was not included in this pilot study.

All students were asked to complete a 20-item multiple-choice test before (pre-test), directly after the block course (post-test 1), and then three months later at the end of the semester (post-test 2). For every item one correct and four wrong answers were given. Tests were created by four independent clinical specialists without knowledge about the content of NESTOR. All tests were anonymized using code names. Students were asked to tick a box if they had used NESTOR for learning and preparation during the semester. Those who did were regarded as “user”, those who did not as “non-user”. At the end of semester students had to pass a mandatory objective structured clinical examination (OSCE) with taking patients history, performing a physical examination and diagnosing actor-patients. The results of the OSCE were taken as evaluation of practical examination skills. Additionally, students’ opinion about the course was evaluated anonymously. As in the written tests (see above) students were asked to tick a box, if they had used NESTOR during the semester and to continue with different questionnaires for NESTOR users and non-users (5-points Likert scale or self-response short answers):

1. Users were asked for (1) their use of NESTOR during the study and also their prior use of the LMS Blackboard to get an idea of the experiences with electronic media and e-learning in general, (2) efficiency of use and structure of NESTOR, (3) satisfaction with its contents and technique, (4) general information concerning the course, and (5) personal information (20 items – 17 Likert scale, 3 short answers).

2. Non-users were asked for (1) their use of the LMS Blackboard, (2) general information concerning the course, (3) reasons for not having used NESTOR and general acceptance of e-learning, (4) personal information (14 items – 12 Likert scale, 2 short answers).

Results of Likert-scaled questions were tabulated and free text answers were reviewed for recurring topics by two reviewers independently.

Written informed consent was obtained from all participants including the allowance to use test and evaluation results as anonymous data for the study (regarding students) and to use a picture of the video for publication (regarding the “actors” of the video, both medical doctors). Additionally, permission was obtained from the responsible educational Ethikkommission der Charité - Universitätsmedizin Berlin.

### Statistical analysis

Written tests were validated by calculating Cronbach’s Alpha. Data of the written tests were analyzed for changes between pre- and post-tests as well as post-test 1 and 2 within the groups of users and non-users using unpaired student’s t-test. To detect differences in the evaluation between NESTOR users and non-users a chi-square test was performed for each question. A p-value less than 0.05 was considered to indicate a significant (< 0.01: highly significant) difference between the observations and the expectations based on the null-hypothesis. Statistical analysis was performed with SPSS® 17.0 statistics software (SPSS Inc., Chicago, IL, USA) and GraphPad Prism®5 (GraphPad Software Inc., San Diego, Ca, USA).

## Results

**Figure 2:**
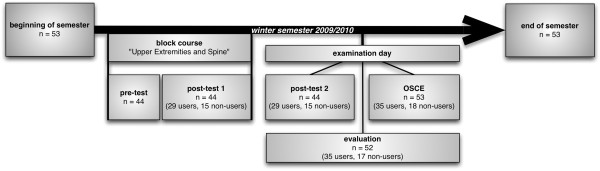
**Flowchart with study design and number of participants.** Legend: Detailed presentation when tests, OSCE and evaluation were performed during the semester and how many students participated (n). The number of NESTOR users and non-users are shown in brackets.

A flowchart illustrating the flow of the study, the number of participants (in each group) and the drop-outs is shown in Figure 2. Altogether, 53 students were enrolled in the PBL curriculum and thus the above mentioned course in winter semester 2009/ 2010. Of these, 52 students (98%) took part in the evaluation (35 NESTOR users (10 male (29%) and 25 female (71%), 17 non-users (7 male (41%) and 10 female (59%)). 44 students (83%) voluntarily participated in the written tests (29 (66%) users, 15 (34%) non-users). All students took part in the OSCE (35 (66%) users, 18 (34%) non-users). Varying participation numbers were due to absence on test-days or single refusals to participate.

### Feedback from evaluation questionnaires

Evaluation of the questionnaires (Tables 1 and 2) showed no significant difference between the two groups with respect to employment status (12 non-users (70%) and 23 users (66%) reported having jobs in addition to studying).

**Table 1:** Results of Likert-scaled evaluation questionnaires filled in by 35 (100%) users (with absolute students’ numbers)

	**Abstain**	**Strongly disagree**	**Disagree**	**Neither agree nor disagree**	**Agree**	**Strongly agree**
**The following Likert-scaled questions/statements were asked…**	**% (n)**	**% (n)**	**% (n)**	**% (n)**	**% (n)**	**% (n)**
…e-learning provides a more flexible learning experience	0 (0)	0 (0)	2.9 (1)	5.7 (2)	37.1 (13)	54.3 (19)
… Managing my free time is a clear advantage of e-learning compared to only face-to-face teaching	0 (0)	2.9 (1)	5.7 (2)	20.0 (7)	37.1 (13)	34.3 (12)
…I estimate my learning success to be high	14.3 (5)	0 (0)	2.9 (1)	40.0 (14)	42.9 (15)	0 (0)
…I was very satisfied with the learning resources on NESTOR	0 (0)	0 (0)	0 (0)	8.6 (3)	68.6 (24)	22.9 (8)
…I estimate an online-support (e.g. via Email) would be very helpful and desirable	2.9 (1)	2.9 (1)	2.9 (1)	22.9 (8)	37.1 (13)	31.4 (11)
…NESTOR is easy to use and well structured	0 (0)	0 (0)	2.9 (1)	14.3 (5)	57.1 (20)	25.7 (9)
…I appreciate the blended learning concept	0 (0)	0 (0)	0 (0)	2.9 (1)	42.9 (15)	54.3 (19)
…learning with NESTOR was fun	0 (0)	0 (0)	2.9 (1)	5.7 (2)	51.4 (18)	40.0 (14)
…NESTOR better prepared me to face clinical problems	0 (0)	0 (0)	2.9 (1)	22.9 (8)	54.3 (19)	20.0 (7)
…NESTOR should continue to be used in future offerings of this course	0 (0)	0 (0)	0 (0)	8.6 (3)	31.4 (11)	60.0 (21)
…The learning texts were easy to comprehend	0 (0)	0 (0)	0 (0)	2.9 (1)	57.1 (20)	40.0 (14)
…Pictures and videos used promoted learning of the material	0 (0)	0 (0)	0 (0)	2.9 (1)	37.4 (13)	60.0 (21)
	Abstain % (n)	Yes % (n)	No % (n)			
… Do you use e-learning offerings on the LMS Blackboard in general?	0 (0)	91.4 (32)	8.6 (3)			
… Do you have a job in addition to studying?	0 (0)	65.7 (23)	34.3 (12)			

**Table 2:** Results of Likert-scaled evaluation questionnaires completed by 17 (100%) non-users (with absolute students’ numbers)

**The following Likert-scaled questions/statements were asked…**	**Abstain**	**Strongly disagree**	**Disagree**	**Neither agree nor disagree**	**Agree**	**Strongly agree**
	**% (n)**	**% (n)**	**% (n)**	**% (n)**	**% (n)**	**% (n)**
… I estimate my learning success to be high	0 (0)	0 (0)	5.9 (1)	58.8 (10)	35.3 (6)	0 (0)
… the course better prepared me for facing clinical problems	0 (0)	0 (0)	0 (0)	29.4 (5)	70.6 (12)	0 (0)
…e-learning in general is useful	11.8 (2)	0 (0)	0 (0)	17.7 (3)	29.4 (5)	41.2 (7)
…e-learning should be offered as a supplement to face-to-face teaching	5.9 (1)	0 (0)	0 (0)	17.7 (3)	35.3 (6)	41.2 (7)
…I have the technical requirements to use e-learning	5.9 (1)	0 (0)	0 (0)	11.8 (2)	5.9 (1)	76.5 (13)
… e-learning gives me a more flexible learning experience	5.9 (1)	0 (0)	5.9 (1)	23.5 (4)	35.3 (6)	23.5 (4)
… Managing my free time is a clear advantage of e-learning compared to only face-to-face teaching	5.9 (1)	0 (0)	0 (0)	23.5 (4)	41.2 (7)	29.4 (5)
	Abstain % (n)	Yes % (n)	No % (n)			
… Do you use e-learning offerings on the LMS Blackboard in general?	0 (0)	58.9 (10)	41.2 (7)			
… Do you have a job in addition to studying?	0 (0)	70.6 (12)	29.4 (5)			

Students who used NESTOR were very satisfied with its offering (92%), approved its overall structure (83%), and had fun learning with it (91%). The blended learning concept was very positively accepted (97%) and NESTOR was considered to be helpful in preparing for clinical situations (74.3%). This very positive evaluation went along with a strong support by the students for continuing the use of this approach (91%). However, non-users also had positive attitudes towards e-learning in general (71%), and were in favor of e-learning being offered as a supplement to face-to-face teaching (77%).

A significant difference was seen in the correlation between the use of NESTOR and pre-existing use of the LMS Blackboard. 91% of NESTOR users were already using Blackboard, compared to 59% of non-users (p < 0.01).

When asked what they liked most about NESTOR, students rated videos first, followed by (in decreasing order) interactive x-ray cases, online-layout and extent of the offered material, and finally availability of podcasts and tests. Users also rated positively that online-contacts were reliable and that their questions were answered promptly. As improvements, students especially suggested more radiographic cases, with single remarks for more theoretical information and anatomy basics without pathologies. As reasons for not having used NESTOR, non-users mentioned a lack of time, not having been informed, not using e-learning in general (preferring books), or having little interest in the subjects of traumatology and orthopedics. When these students were asked what would make it more likely for them to use the e-learning offer, they especially mentioned more announcements to be helpful for the future.

Both users and non-users showed in general a strong acceptance of a blended learning concept. Asked, which aspects of orthopedics and traumatology could better be represented by e-learning versus face-to-face lessons, all answering students (11 users, 3 non-users) agreed on e-learning to be more useful for theoretical contents and as preparation for specific skills, for example, when exploring radiology-cases, or introducing physical exam or surgical procedures with videos. Face-to-face lessons were seen as particularly helpful for practicing examination skills or having discussions about clinical and radiological cases. A typical statement of one student was “I like the combination of both. First you would use e-learning for getting to know the contents and for self-training. Then you would practically train the gained knowledge in the lessons with a teacher”.

### Written tests and OSCE results

**Figure 3:**
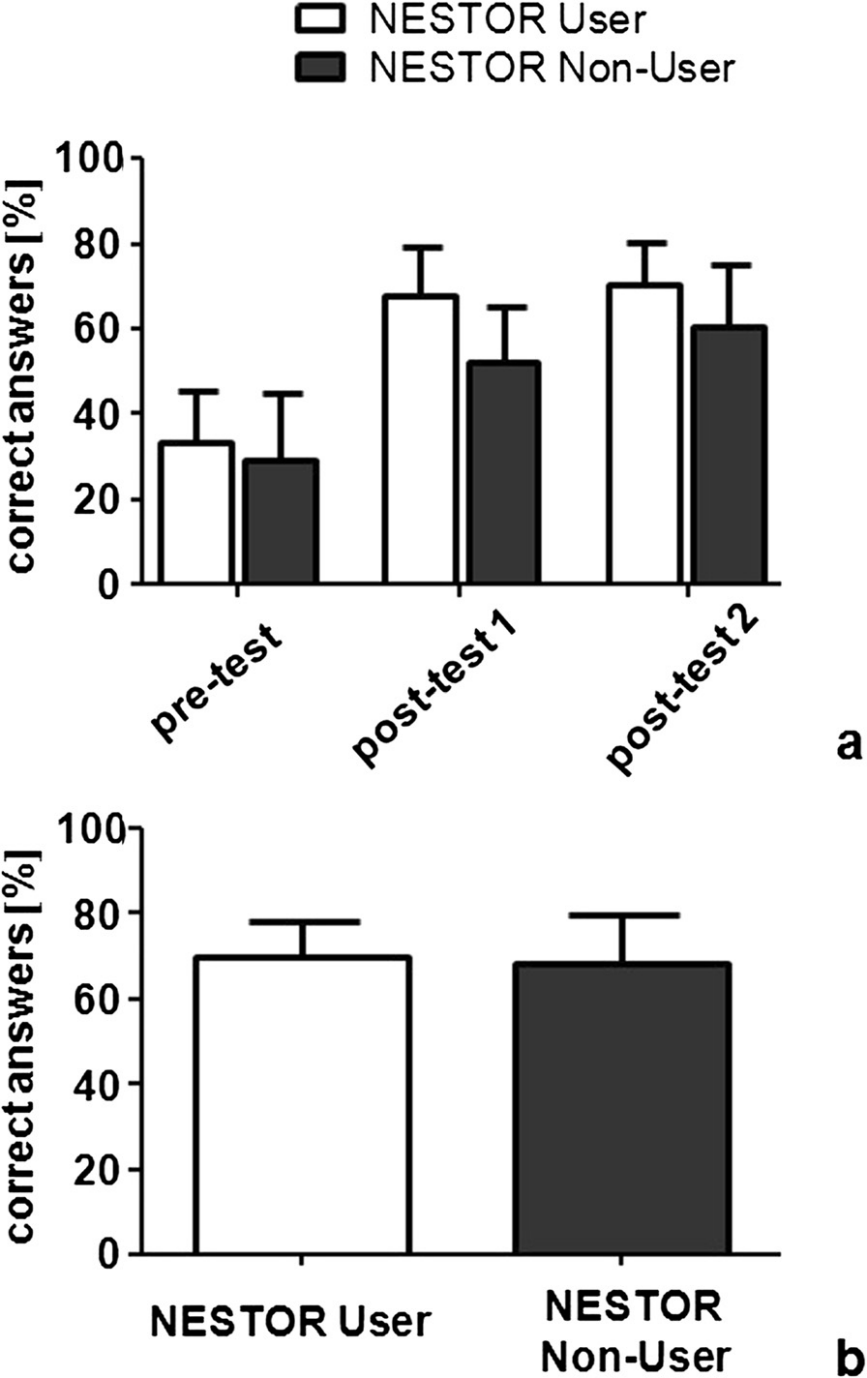
**Results of written tests and OSCE.** Legend: **(a)** The results of the written pre-test, post-test 1 and post-test 2 for users and non-users of NESTOR showed significant improvement in post-test 1 and 2 for both groups, compared to the pre-test (p < 0.001). NESTOR users further improved significantly from post-test 1 to 2 (p = 0.009), whereas non-users did not. **(b)** The Results of the objective structured clinical examination (OSCE) showed no differences comparing users and non-users of NESTOR. Whisker = standard deviation.

Cronbach’s Alpha for the written tests were 0.62 (pre-test), 0.64 (post-test 1), and 0.63 (post-test 2). Results of the pre-test and both post-tests for the groups of users and non-users of NESTOR are shown in Figure 3a. Pre-tests revealed no differences between the groups. Users as well as non-users had significantly better results in both post-tests (p < 0.001), with slightly better results for NESTOR users in both post-tests. Students who used NESTOR further improved their results significantly between post-test 1 and 2 (p = 0.009), whereas non-users did not. The results of the OSCE revealed no differences between the groups (Figure 3b).

## Discussion

The purpose of this pilot study was to give first impressions of the effect of a blended learning concept in orthopedics and traumatology called NESTOR both on students’ satisfaction and on its contribution to acquisition of knowledge and clinical skills in a problem-based learning curriculum, which already provides an intensely practice-oriented teaching environment. To the best of the author’s knowledge, this is the first study examining the influence of blended learning not only on satisfaction, but also on knowledge and practical clinical skills of students in traumatology and orthopedics, two highly practically oriented medical subjects.

Evaluations of students’ opinion and acceptance can be seen as first step when establishing a new e-learning program [4,14,21]. Consistent with the literature, this study revealed a high approval of the participating users for the additionally offered e-learning contents. While a broad acceptance is crucial for successful e-learning implementation [1], it is also important to evaluate its influence on students’ gain of knowledge and skills [9].

Thus, as second step, not only the impact on users’ satisfaction, but also on their knowledge should be demonstrated [6,21,22]. For this pilot study we have chosen newly developed written tests to evaluate improvement in theoretical knowledge, which seem to be valid as indicated by the measured Cronbach’s Alpha values. While some studies showed benefits of e-learning on improvement of students’ knowledge [6,23] others did not – despite of positive evaluation [2,8,22]. In this pilot study, we found a significant improvement from pre- to post-tests for both groups. NESTOR users scored better in the written post-tests than non-users and showed further improvement between post-test 1 and 2. A possible interpretation for this success in the group of NESTOR users may be students’ very positive attitude towards e-learning and a high satisfaction with structure and contents. However, these results should be interpreted carefully, especially when referring this effect exclusively to the use of NESTOR. In a recent review, Rowe et al. [12] showed that the existing data to evaluate an improvement of clinical competencies by blended learning can still be regarded as rudimentary. It seemed to be a problem of the study design in a clinical environment to determine the effect of blended learning exclusively. Rowe et al. identified 71 studies dealing with the role of blended learning in the clinical education of healthcare students, but only 7 articles were enrolled for the review due to methodological flaws of the remaining 64.

As potential third step it might be anticipated that even practical clinical skills may be improved through blended learning in this context [9], which has been shown in some studies [9,16,24]. The results of the OSCE revealed no differences between users and non-users in this pilot study with high scores in both groups. These findings are consistent with other studies which failed to detect significant benefits on examination performance or other practical clinical skills with e-learning implementation [21,25,26]. A possible explanation could be that the pre-existing, highly clinically oriented curriculum made it difficult for any additional e-learning exposure to further improve skills.

In this context, the question may arise what e-learning potentially can achieve [1]. It can be argued that clinical examination skills will always be preferentially based on personal experiences and training rather than on the use of e-learning – unlike acquiring skills in other areas such as radiological diagnosis [16]. In the presented pilot study, e-learning enhanced competencies for gaining theoretical medical knowledge. Further research will be necessary to determine, if it is possible to adjust the components of a blended learning approach in this context to achieve also an improvement of practical skills compared to mere face-to-face teaching. However, as knowledge about diseases is an important basis for developing treatment and examination skills, this and the overall high approval provide good arguments for the continued use of NESTOR in the preparation for the tested subjects. Following suggestions of the non-users, acceptance of the program might be further increased by improving announcements about it. Additionally, it could be made even more appealing with links to e-learning programs of other subjects (e.g. anatomy) of the faculty.

Concerning the willingness to use e-learning offerings voluntarily, an additional inference can be taken from this study en passent: As the use of NESTOR was significantly linked to the use of LMS Blackboard, the likelihood of voluntarily using an e-learning offering may be directly connected to the acceptance and use of the hosting LMS. This would require the need for the entire faculty to join in a combined effort to establish e-learning offerings broadly to increase students’ familiarity with such resources. Thereby, voluntary and perhaps even mandatory use of e-learning components could be increased.

There are some limitations to be noted in this study. A selection bias cannot be excluded due to the voluntary nature of participation and use of NESTOR, also with respect to the significant correlation between this aspect and a pre-existing use of the LMS Blackboard. This can be seen as main shortcoming, which was tolerated because data evaluation was incorporated into an ongoing mandatory course to establish this new blended learning concept. This pilot study design guaranteed a high practical orientation and a sufficient number of participants. However, for final conclusions on the chosen blended learning technique a randomized controlled trial should rather be performed in the future. Furthermore, preexisting experiences in students’ physical examination skills will have to be evaluated in the final implementation study by a pre-test. The possibility that the exposure to or interaction with any additional resources or experiences led to an increase in knowledge (i.e. due to a possibly more in-depth coverage of the topic), cannot be excluded completely with the study design. In a future study, there should also be a tracking function to detect the individual accessed e-learning tools or the time students spend with them. Such a study design would also avoid the present division of users and non-users merely according to their own declaration, as this step does not guarantee a correct assignment of data to the test and control group, respectively.

Finally, no direct correlation between test results and questionnaires was possible due to guaranteed complete anonymity. This could have potentially given more information about individual user’s attitudes towards the program and their concomitant test results.

## Conclusions

This pilot study could underline that it is possible to achieve an improvement in theoretical knowledge combined with high acceptance of students with a blended learning program. The results indicate that the blended learning concept might be superior compared to face-to-face teaching alone, even in the setting of a problem-based learning environment where a high level of self-reliant learning has already existed. Future research on the presented concept should assess which blended learning scenarios might support best students’ acquisition of practical examination skills and identify further crucial points in knowledge and competence transfer to support the improvement of teaching in this context. Therefore well designed randomized controlled trials within realistic clinical education scenarios are still needed.

## Competing interests

The authors declare that they have no competing interests.

## Authors’ contributions

DB and GS designed the study. NH, DB, AA and KS collected the data. DB, NH and EH analyzed and interpreted the results. DB drafted the article with substantial support by AA and EH. NH, KS and GS revised the paper critically. All authors approved the submitted version to be published.

## Pre-publication history

The pre-publication history for this paper can be accessed here:

http://www.biomedcentral.com/1472-6920/14/17/prepub
